# Error rate on the director's task is influenced by the need to take another's perspective but not the type of perspective

**DOI:** 10.1098/rsos.170284

**Published:** 2017-08-16

**Authors:** Edward W. Legg, Laure Olivier, Steven Samuel, Robert Lurz, Nicola S. Clayton

**Affiliations:** 1 Department of Psychology, University of Cambridge, Cambridge CB2 3EB, UK; 2 UFR Science de la Vie et de l'Environnement, Université de Rennes 1, Rennes, Bretagne, France; 3 Brooklyn College, CUNY, Brooklyn, NY, USA

**Keywords:** theory of mind, perspective-taking, Level 2 perspective-taking, director's task

## Abstract

Adults are prone to responding erroneously to another's instructions based on what they themselves see and not what the other person sees. Previous studies have indicated that in instruction-following tasks participants make more errors when required to infer another's perspective than when following a rule. These inference-induced errors may occur because the inference process itself is error-prone or because they are a side effect of the inference process. Crucially, if the inference process is error-prone, then higher error rates should be found when the perspective to be inferred is more complex. Here, we found that participants were no more error-prone when they had to judge how an item appeared (Level 2 perspective-taking) than when they had to judge whether an item could or could not be seen (Level 1 perspective-taking). However, participants were more error-prone in the perspective-taking variants of the task than in a version that only required them to follow a rule. These results suggest that having to represent another's perspective induces errors when following their instructions but that error rates are not directly linked to errors in inferring another's perspective.

## 1. Introduction

To make sense of our everyday conversations, we often need to account for differences between our own and another's perspective. However, adults often overestimate the extent to which their knowledge is shared by others. Thus, adults are prone to producing egocentric errors during conversations and will refer to information that is not known to another, even if this information was meant to be secret, and will respond to others' instructions based on privileged information and not shared knowledge [1–4].

Models of how instructions from different perspectives are interpreted suggest a tripartite process involving (i) inferring the other's perspective, (ii) holding that perspective in mind, and (iii) using the information from that represented perspective [5,6]. Thus, an erroneous response to another's instruction may occur because of a failure of one or more of these components [6]. Firstly, participants may fail to infer another's perspective, that is to say they fail to represent or misrepresent what the other individual knows or sees. Secondly, participants may fail to remember the representation they inferred. Finally, participants may fail to *use* the information present in their, correctly inferred, representation of the other's perspective when selecting how to respond to the other individual's instruction.

Previous research indicates that failures when interpreting instructions are related to inferring another's perspective. In a key test of why these failures occur, one group of participants were instructed to click on objects that a ‘director’ could see; while a second group of participants were instructed to follow a rule to click on objects that were not in front of an opaque barrier [1]. Correct responses were the same for both variants of the task, because the objects that the director could see were de facto objects that were not in front of an opaque barrier. Importantly, both versions of the task required participants to store a key piece of information (the director's perspective or the rule) and to use that information when responding to the instruction. However, only the perspective-taking variant required participants to infer what the director could or could not see. Crucially, participants in the perspective-taking group made more errors than participants in the rule-based group, indicating that error rates are linked to the need to infer another's perspective.^1^

However, it remains unclear *why* error rates increase when participants need to infer another's perspective. Three plausible explanations exist. Firstly, participants might make errors in their inferences—i.e. an error in step (i) of the tripartite process—and these errors are carried over when storing and using this information. Secondly, storing and using an inferred representation may be harder than storing and using a rule—i.e. an error at step (ii) or (iii) that is a consequence of the outcome of step (i). Thirdly, previous research has shown that error rates are linked to extraneous cognitive demands, and it may be that the extra cognitive demand of having to make an inference increases participants’ error rates on such tasks [7]—i.e. an error that is indirectly caused by step (i) being a cognitively demanding process.

The current experiment tested the first of the three hypotheses presented—namely, whether errors when interpreting other's instructions are the result of errors in inferring the other's perspective. We investigated this hypothesis by manipulating the nature and complexity of the perspective that had to be inferred. If error rates are due to errors in inferring others' perspectives, then participants should make more errors when that inference is harder. In addition, we sought to reproduce evidence that perspective-taking leads to more errors than following a task-equivalent rule. To manipulate the complexity of the perspective to be taken, we drew on the distinction between Level 1 perspective-taking—the ability to understand *what* someone can or cannot see—and Level 2 perspective-taking—the ability to understand *how* someone sees things [8–10]. The nature of Level 1 perspective-taking is binary (the item is either seen by another or not) and may lend itself to fast and efficient processing, but Level 2 perspective-taking is considered more cognitively taxing because it requires computing *how* an object appears to another person [11,12]. This distinction is evident in the developmental literature where Level 2 perspective-taking is considered to develop later than Level 1 perspective-taking [8,13,14]. In addition, it is thought that Theory of Mind (i.e. the ability to attribute mental states to others) is a necessary component of Level 2 perspective-taking, but it may not be of Level 1 perspective-taking, making the latter ability less cognitively demanding than the former. Specifically, performance in Level 1 perspective-taking tasks may be, at least partially, underpinned by a relatively simple cognitive system that processes lines of sight without processing the mental state of ‘seeing’ [11,15]. Consequently, the hypothesis that egocentric errors that occur when following instructions are a result of failing to infer a perspective would predict an increase in errors for tasks requiring Level 2 perspective-taking relative to tasks requiring Level 1 perspective-taking. However, if error rates occur because it is difficult to store and/or use information from a representation, then there should be no difference in error rates between our Level 1 perspective-taking and the Level 2 perspective-taking tasks. Similarly, if the cognitive demands of having to make an inference per se are the cause of errors, then there should be no difference between participants' performance on the Level 1 and Level 2 variants of our task.

## 2. Material and methods

Following Apperly *et al.* [1], three versions of an instruction-following task (henceforth, ‘the director's task’) were run to compare errors when reasoning about Level 1 perspectives (L1 task) and Level 2 perspectives (L2 task) and when performing a non-social visual discrimination (Rule task).

### 2.1. Participants

A total of 90 adults were recruited for the study using the online participant platform Prolific Academic (www.prolific.ac) and they responded to the test entirely online. Thirty adults (9 male, 21 female; mean age: 35 years, range: 18–53) were recruited for the L1 task, all of whom successfully completed a manipulation check at the end of the experiment to ensure they understood the effect of the barriers used in the task. Thirty adults (16 male, 14 female; mean age: 36 years; range: 19–64) were recruited for the L2 task but five individuals were excluded from the final analysis: four for failing the final manipulation check and one for indicating that they were colour-blind. Thirty adults were recruited for the Rule task (15 male, 15 female; mean age: 36 years; range: 19–64) but six individuals were excluded from the final analysis: five for failing the manipulation check and one for indicating that they were colour-blind. All participants were native English speakers who received monetary compensation for taking part and no individual took part in more than one task.

### 2.2. Materials

**Figure 1.**
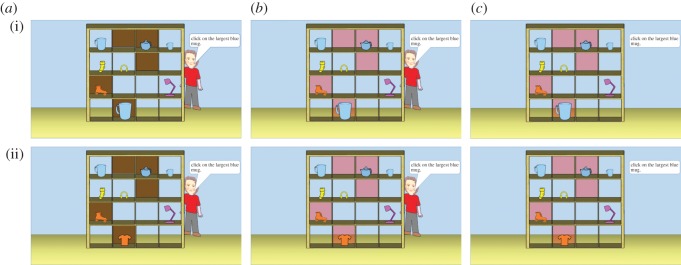
Example stimuli from relational trial types for Experimental (row i) and Control trials (row ii). The columns depict which task participants received (*a*) L1 task, (*b*) L2 task and (*c*) Rule task.

Computer-generated pictures of a four by four set of shelves (creating 16 slots that could contain objects) were used as stimuli (figure 1). A protagonist (the ‘director’), was depicted on the far side of the shelves for the L1 task and L2 task in which the director's instructions to the participants were presented in a white speech bubble in the upper right corner of the picture. For the Rule task, the director was removed and the instructions were placed in a rectangular white box. To manipulate what the director could or could not see in the L1 task, five slots had opaque occluders. To manipulate *how* the director saw objects in the slots, the L2 task shelves had red-translucent barriers—these barriers ensured that how the items in the slots appeared to the director was altered in a manner consistent with subtractive colour mixing with the barriers making yellow objects appear orange and blue objects appear purple. Red-translucent barriers were also used in the rule version.^2^ Sixteen unique layouts of the barrier positions were created and, for each layout, eight objects were presented on the shelves. The same layouts and objects were used in all three versions of the task.

### 2.3. Procedure

At the start of each task, an example of the shelves from the participant's and the director's perspective was presented to clarify the effect of the opaque or translucent barriers (see electronic supplementary material, figure S2).

A single trial involved the participant being presented with a single written instruction (either in a speech bubble or rectangle) that referred to one of the items on the shelves.

Over the course of the experiment, participants were presented with 32 different arrays of objects in the slots and were presented with three instructions per array (a total of 96 trials). Two instructions per array were ‘filler’ instructions that did not require perspective-taking as they uncontestably referred to a specific item in the shelves; as in previous studies, performance on these filler trials were not analysed because participants were expected to perform at ceiling. One instruction per array was a critical instruction; for half of these instructions, the experimental trials, the correct response required that participants took the director's perspective or followed the rule because an object that fitted the description in the instruction (the *distractor object*) was in a slot with a barrier. In the other half of the arrays, participants were presented with control instructions; the instructions and the arrays were matched to those presented during experimental instructions except that the distractor object was replaced by another object that could not be mistaken for the object of the director's instruction. For example, in experimental instructions participants would be asked to ‘Click on the largest blue mug’ and the array would contain three blue mugs of different sizes with the largest mug being in a slot with an opaque barrier. By contrast, for control instructions the array would be the same but the largest mug would be replaced with a t-shirt (see figure 1 for a depiction of the stimuli used for experimental and control instructions in all three versions of the experiment).

The presentation order of the grids was pseudo-randomized such that the matched *control arrays* and *experimental arrays* were presented 16 arrays apart. Half of participants received one order of trials and the other half received the reverse order.

Following Experiment 3 of Apperly *et al*. [1], *experimental instructions* were divided into two types (eight trials per type) *ambiguous instructions* (figure 1*a*) and *relational instructions. Ambiguous instructions* were those that involved a distractor object exactly like the correct object except that it was behind a barrier e.g. participants would be instructed to ‘Click on the yellow ball’ when there were two yellow balls, one behind a barrier and one in an open slot); and *relational instructions* where the distractor object which, notwithstanding perspective-taking and rule-following considerations, was the best match for the instruction except for being behind a barrier (e.g. participants would be instructed to ‘Click on the smallest blue ball’ but the smallest ball was in a slot with a barrier).^3^ In the original versions, participants were also asked to move the objects to the left and right but we did not include this in our study because this invokes a form of Level 2 perspective-taking because participants need to reason about how their left is actually the director's right.

To ensure that we only used the data from participants who had understood the effect of the barriers and who had maintained that understanding to the end of the experiment, participants were required to perform a manipulation check at the end of the experiment. This involved them being presented with an array filled with the numbers 1–16, with each number in a different slot (see electronic supplementary material, figure S2). Five numbers were behind the barriers of the type used in the version of the task participants had received. For the L1 task, participants were asked to click on the slots that the director could not see. For the L2 task, the numbers were coloured either yellow and blue and the participants were asked to click on the numbers that would appear purple or orange to the director. Only data from those participants who clicked on all five numbers in the slots with barriers were used in the analysis.

### 2.4. Analysis

Following Apperly *et al*. [1], we recorded whether participants clicked on the correct object, the distractor object or an alternative object. Our main analysis focused on the number of clicks on the distractor object during the *experimental instructions* for *experimental arrays,* henceforth egocentric errors*.* These errors are typically analysed with ANOVAs (e.g. [1,16]); however, this is an inappropriate technique for this type of count data because confidence intervals can include impossible events (e.g. intervals extending below 0) and data tend not to fit the homogeneity assumptions of an ANOVA [17]). Given that the baseline error rate (error rate if participants never take into account another's perspective or the rule) in ambiguous trials is 50% and in relational trials it is 100%, we conducted separate analyses for each trial type (note that, in line with previous studies, we found that, collapsed over all trial types, participants are more prone to errors on relational trials than on ambiguous trials: Wilcoxon signed-rank test, *W* = 449, *p *< 0.001). To compare error rates between the L1 task, L2 task and Rule task, data were analysed using a Kruskal–Wallis test. Planned comparisons were performed using Mann–Whitney *U*-tests to compare the L1 task to the Rule task, the L2 task to the Rule task and the L1 task to the L2 task. Previous studies found that error rates were greater in the perspective-taking variants of the experiment than in the rule variant. Thus, error rates were expected to be higher in both the L1 task and L2 task than in the Rule task. Critically, if the complexity of the perspective to be taken affects errors, we expected more errors in the L2 task than in the L1 task.

## 3. Results

Overall, participants responded to instructions based on the director's perspective or the rule and made few errors in experimental trials. In the L1 task, the mean error rate was 16.7%; in the L2 task, it was 10.0%; and in the Rule task, it was 3.9%. Of these, the vast majority of errors were clicks on the *distractor object* (egocentric errors as a percentage of total trials: L1 task 15.8%, L2 task 10.0% and Rule task 3.1%). During the control trials, participants never clicked on the object that had replaced the distractor object.

### 3.1. Ambiguous trials

The number of egocentric errors made by participants in the ambiguous trials varied depending on the experimental condition; for these trial types the mean number of errors was 10.42% (s.d. = 20.78%) in the L1 task, 3.50% (s.d. = 13.27%) in the L2 task and 1.56% (s.d. = 7.65%) in the Rule task (Kruskal–Wallis test, $\chi_{\left(2\right)}^{2}=6.49$, *p = *0.039). More egocentric errors were made in the L1 task than in the Rule task (Mann–Whitney *U*-Test: *W* = 441, *p *= 0.03). However, we found no significant difference between the error rates in the L2 task and the Rule task (Mann–Whitney *U*-Test: *W* = 311.5, *p *= 0.58). Moreover, error rates were similar across the two perspective-taking versions of the task (Mann–Whitney *U*-Test: *W* = 444, *p *= 0.083).

### 3.2. Relational trials

The number of egocentric errors made by participants varied depending on the experimental condition for the relational trials; for these trial types the mean number of errors was 21.25% (s.d. = 33.66%) in the L1 task, 16.50% (s.d. = 28.80%) in the L2 task and 4.69% (s.d. = 20.4%) in the Rule task (Kruskal–Wallis test, $\chi_{\left(2\right)}^{2}=10.11$, *p = *0.006). A greater number of egocentric errors were made in relational trials for both perspective-taking tasks (L1 task and L2 task) compared with the Rule task (Mann–Whitney *U*-Test: L1 task versus Rule task: *W* = 506.5, *p = *0*.*002 ; L2 task versus Rule task: *W* = 405, *p *= 0.007). Error rates in relational trials between the two perspective-taking tasks did not differ (Mann–Whitney *U*-Test: *W* = 397.5, *p *= 0.678).

## 4. Discussion

In the current study, we investigated whether or not performance in the director's task was influenced by the type of perspective-taking necessary to interpret an instruction. We replicated the results of previous studies that have demonstrated that error rates are higher when participants are required to account for another's perspective compared to when participants follow an arbitrary rule that requires the same behavioural response as in the perspective-taking scenario [1]. Furthermore, we found that participants' error rates were not influenced by the complexity of inferring the instructor's perspective. Specifically, participants were similarly error-prone when having to take into account *how* an object appears to the instructor (Level 2 visual perspective-taking) compared to when they only had to take into account whether or not that object could be seen by the instructor (Level 1 visual perspective-taking).

Previous studies have indicated that error rates on the director's task are linked to the need to infer an alternative perspective. We corroborate that finding by demonstrating that participants tend to be less error-prone when following rules than when perspective-taking is necessary. However, the difference in the number of egocentric errors made when following a rule and when taking a perspective appears to be specifically linked to relational trials. In the current study, we found that for both perspective-taking versions (L1 and L2) of the task participants were more error-prone on relational trials than on relational trials in the rule version of the task. This was not the case for ambiguous trials. Although participants were more error-prone on these trials for the L1 version of the experiment compared with the rule version, there was no difference in performance on ambiguous trials between the L2 version and the rule version of the experiment. These differences in the pattern of results between ambiguous and relational trial types are not unique to our experiment; Apperly *et al.* [1] also found a similar difference between the ambiguous and relational trial types, with participants showing greater accuracy in relational trials on the rule version of the task than on the perspective-taking version, and similar accuracy levels in both versions on ambiguous trials. It remains to be determined why performance on ambiguous trials appears to be relatively uninfluenced by whether the director's task requires following a rule or perspective-taking or why, unlike Apperly *et al.* [1], we found a difference in performance on ambiguous trials between the L1 task and a rule-based task. These results could be influenced by a ceiling effect because error rates on the rule-based task in the ambiguous trials tended to be extremely low (mean = 1.56%). There is also an important difference in terms of the costs of processing the director's instructions between ambiguous and relational trials. In relational trials, the instructions create a direct conflict between responses based on the director's perspective and responses based on the participant's perspective. This conflict is not present for ambiguous instructions where, from the participant's perspective, two objects fit the description with one of these two fitting the description from the director's perspective. Thus, ambiguous trials do not require participants to completely disengage from what object they believe to be the best fit for the instruction, and may, therefore, be less sensitive to detecting egocentrism.

Central to our concerns is the lack of a difference in participants' performance in the L1 and L2 tasks. We had hypothesized that the process of perspective-taking involved in the L1 task should be easier than the equivalent process in the L2 task. One possibility is that the difference in complexity between Level 1 and Level 2 perspective-taking is not as great as we anticipated—but such an interpretation should be treated cautiously because of the well-established developmental progression from Level 1 to Level 2 and because studies show that Level 1 perspectives but not Level 2 perspectives can be encoded rapidly (including results from eye-tracking in the director's task; [18]). Alternatively, it is possible that the differences in the stimuli used in our L1 and L2 tasks may have masked differences in participants’ performance. For instance, red-translucent barriers were used in the L2 task and brown barriers were used in the Level 1 task. It is possible that the red-translucent barriers were more salient than the brown opaque barriers and that this may have aided participants in the L2 task—an interpretation that could be supported by a trend for participants to make fewer errors on ambiguous trials in the L2 task than in the L1 task. However, we believe that any potential difference in the salience of the barriers is unlikely to have masked a difference in error rates because participants must attend to the slot in which they are selecting the object and so are unlikely not to notice the barriers, regardless of their colour. Furthermore, the differences found in error rates on relational trials between the L2 task and the Rule task (where red barriers were used) indicate that the simple presence of red barriers does not negate the specific difficulties participants have when responding based on the director's perspective.

In the introduction, we argued that the results from director's tasks are typically considered in terms of a tripartite process of interpreting another's instruction based on their perspective, a process that involves inferring, then storing and then using a representation of another's perspective. Furthermore, as previously discussed, the difference in error rates between a perspective-taking and rule version of the director's task indicates that errors are linked to the inference component of the process, but this does not tell us how or why the need to make an inference leads to an increase in participants' errors. Previously, we discussed three plausible explanations of why the need to make an inference could influence participants’ error rates. Firstly, participants might make errors in their inferences and these errors are carried over when storing and using this information. Secondly, storing and using an inferred representation may be harder than storing and using a non-inferred rule. Thirdly, the extra cognitive demand of having to make an inference could increase participants’ error rates.

To test the first of these hypotheses, in the current study, we manipulated the complexity of the perspective to be taken by manipulating whether participants had to infer a Level 1 or a Level 2 perspective. This hypothesis proposes that participants make errors in inferring what others can see and that consequently harder to infer states, such as those involved in Level 2 perspective-taking, should lead to more errors. The other two hypotheses remain neutral about the effect of Level 2 perspective-taking on participants' error rates because they suggest that the inference component of the task has an indirect influence on participants’ error rates, namely that its presence induces errors either because of the content of the representation formed by inferring the other's perspective or because inferring another's perspective is cognitively demanding. In line with the idea that the need to make an inference has an indirect influence on participants' error rates, we find that error rates are not linked to how complex the perspective to be inferred is.

The argument that error rates in the director's task are not the direct result of errors in inferring the director's perspective is supported by two additional pieces of evidence. Firstly, participants who have high scores on the Autism-Spectrum Quotient make a similar level of errors to lower-scoring individuals, yet scoring highly on the Autism-Spectrum Quotient should be indicative of a poorer ability to infer perspectives [19]. Furthermore, there is evidence that performing a cognitively demanding secondary task has a detrimental effect on participants' performance in both the perspective-taking and rule-based versions of the instruction-following task [7]. Therefore, cognitive load appears to increase error rates even when inferring that a perspective is not a necessary feature of the task. Taken with our current findings, these results suggest that errors in the director's task do not come about because participants are error-prone when inferring perspectives. Furthermore, the finding that cognitive demands influence both rule-based and perspective-taking variants of the director's task helps to elucidate why, despite the evidence suggesting that participants do not struggle to infer perspectives, error rates are higher on tasks that require participants to infer perspectives compared with rule-following tasks. Specifically, just as a cognitively demanding secondary task produces higher error rates in the director's task, the extra cognitive demand of having to infer a perspective may indirectly lead to higher error rates in the perspective-taking variants. Thus, a combination of the results of previous studies and the current study suggest that the third of our hypotheses, namely that the cognitive demands of inferring perspectives produces errors in the director's task, is the most probably explanation of performance.

Crucially, the suggestion that the cognitive demands of inferring another's perspective give rise to errors on the director's task helps to bring together apparently discrepant results in this field. On the one hand, it has been argued that participants may not need to specifically engage in perspective-taking when participating in the director's task and it is argued that, instead of perspective-taking, participants may adopt an alternative spatial frame when responding—one that is centred on the opposite side of the shelves to the side they face. This is a view that is supported by evidence that reasoning about what a photograph taken from the opposite side of the shelves would depict elicits a similar error rate to reasoning about what a director in the same position would see. This is argued to be evidence that the same mechanism may be involved in attributing perspectives and when performing a non-social task that involves adopting a different spatial framework (e.g. reasoning about the content of a photograph). By contrast, evidence that specifically social manipulations influence performance on the director's task is argued to be evidence that errors are related to a mechanism that processes social stimuli. For instance, participants who undergo counter-mirror training, where they have to perform a different action to another individual, or who come from collectivist cultures outperform controls [20,21]. Crucially, both sets of results can be explained under the cognitive load hypothesis because these results can be explained by similarities or differences in cognitive load. For instance, adopting another's view or adopting an equivalent non-social spatial frame could present similar cognitive demands but differences in participants' propensity to adopt another's perspective induced by culture or experimental manipulations are likely to reduce these cognitive demands. In summary, we found that participants tend to make more errors when following instructions that need to be interpreted by representing another's perspective than when following a rule. Critically, we found that the type of perspective-taking necessary in these tasks does not influence participants' error rates. Thus, our findings lend support to the suggestion that although error rates on these tasks are linked to the need to infer another's perspective, the errors are unlikely to be because participants' inferences about the other's perspective are incorrect. Instead these errors may occur because inferring another's perspective places extra cognitive demands on participants, which makes them more error-prone than when they do not need to infer another's perspective.

## Supplementary Material

Supplemental Figures

## Supplementary Material

Electronic Supplementary Material

## Footnotes

1 There is evidence that participants make equivalent amounts of errors when reasoning about non-mental representations, such as the contents of photographs. This suggests the mechanism used to make these inferences may not uniquely process mental states and perspectives. Regardless of whether the mechanism is specific to mental states or is common to reasoning about representations in general, it is important to establish why errors occur when this type of reasoning is employed.

2 Previous research had shown a distinction between an L1 task and a task that required following a rule. Here, we aimed to ensure that the rule version was matched as closely as possible to the L2 task because no previous studies had made this type of comparison.

3 Note that while our instruction types matched those used by Apperly *et al.* [1], there are some differences in the presentation of these instructions. Firstly, in ambiguous trials we used two objects that were identical (e.g. two blue teapots with one behind a barrier), whereas Apperly *et al.* [1] used two different objects with the same name (e.g. a computer mouse and a cartoon animal). This change was because we needed to use simplistic stimuli with a uniform colour to produce the colour change for the L2 task. Secondly, the instructions provided by the director were in speech bubbles (and not spoken) and were extremely specific, indicating the colour and (in relational trials) the superlative qualities of the object (e.g. smallest or largest teapot). This over-specification was important because it was essential for the L2 task in which the director had to refer to the colour of the object; so we ensured that their instructions consistently over-specified objects by describing their colour in both the control and experimental trials.

## Data accessibility

Data are included in the electronic supplementary material. The three versions of the experiment can be tried online (www.goo.gl/E2JFI7). We are unable to make the raw set-up of the experiment publicly available as it is hosted within the Qualtrics environment (an annotated screen shot of the set-up is available in the electronic supplementary material). Readers with a Qualtrics account can contact us (ewl24@cam.ac.uk) if they would like us to share this set-up with them.
