# Supplementary material for: Error rate on the director's task is influenced by the need to take another's perspective but not the type of perspective
Source: R Soc Open Sci. 2017 Aug 16;4(8):170284. doi: 10.1098/rsos.170284 (PMC5579093; doi:10.1098/rsos.170284)
Supplement: Supplemental Figures [file rsos170284supp1.pptx]

## Slide 1
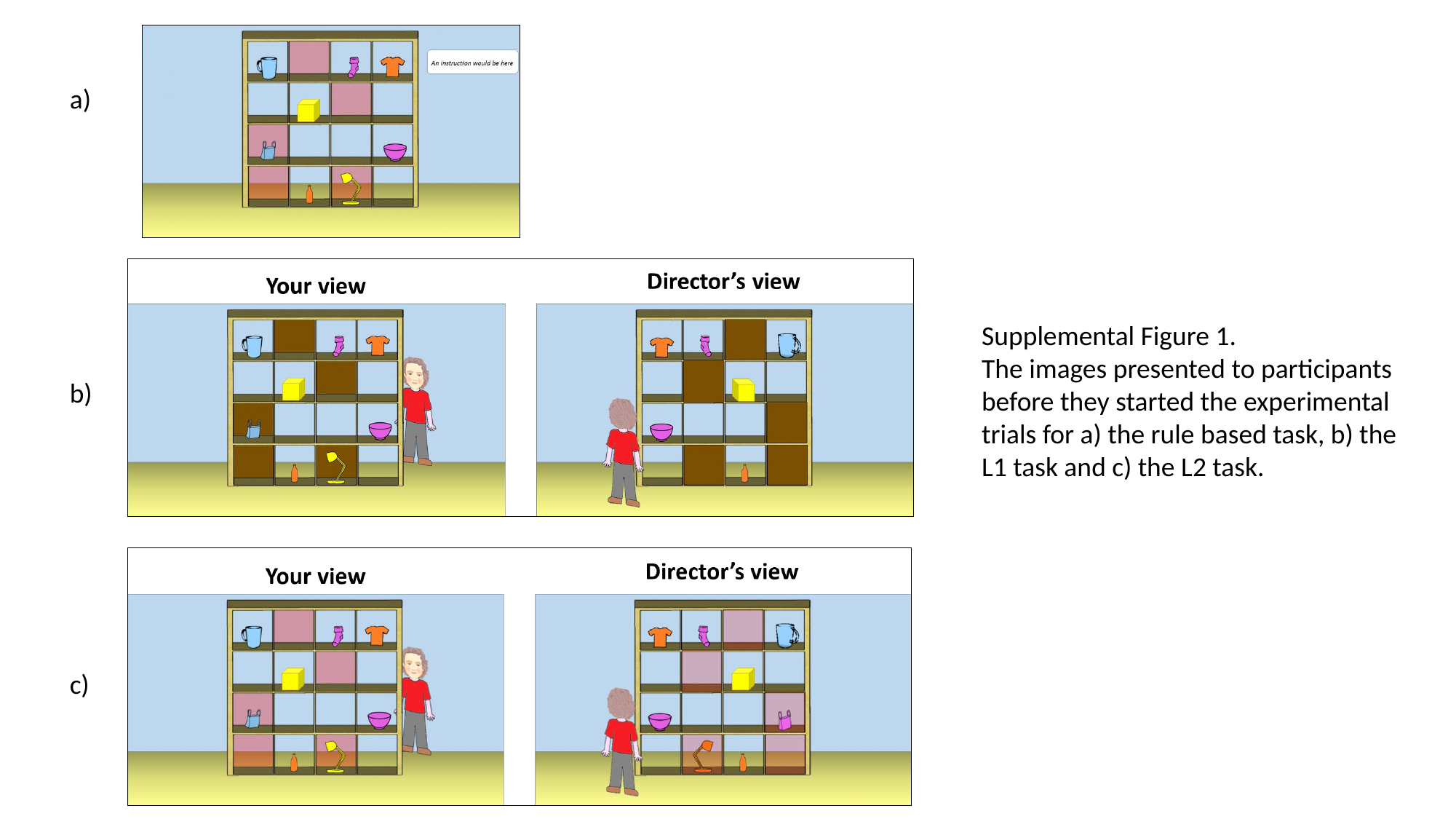

a)
Supplemental Figure 1.
The images presented to participants before they started the experimental trials for a) the rule based task, b) the L1 task and c) the L2 task.
b)
c)

## Slide 2
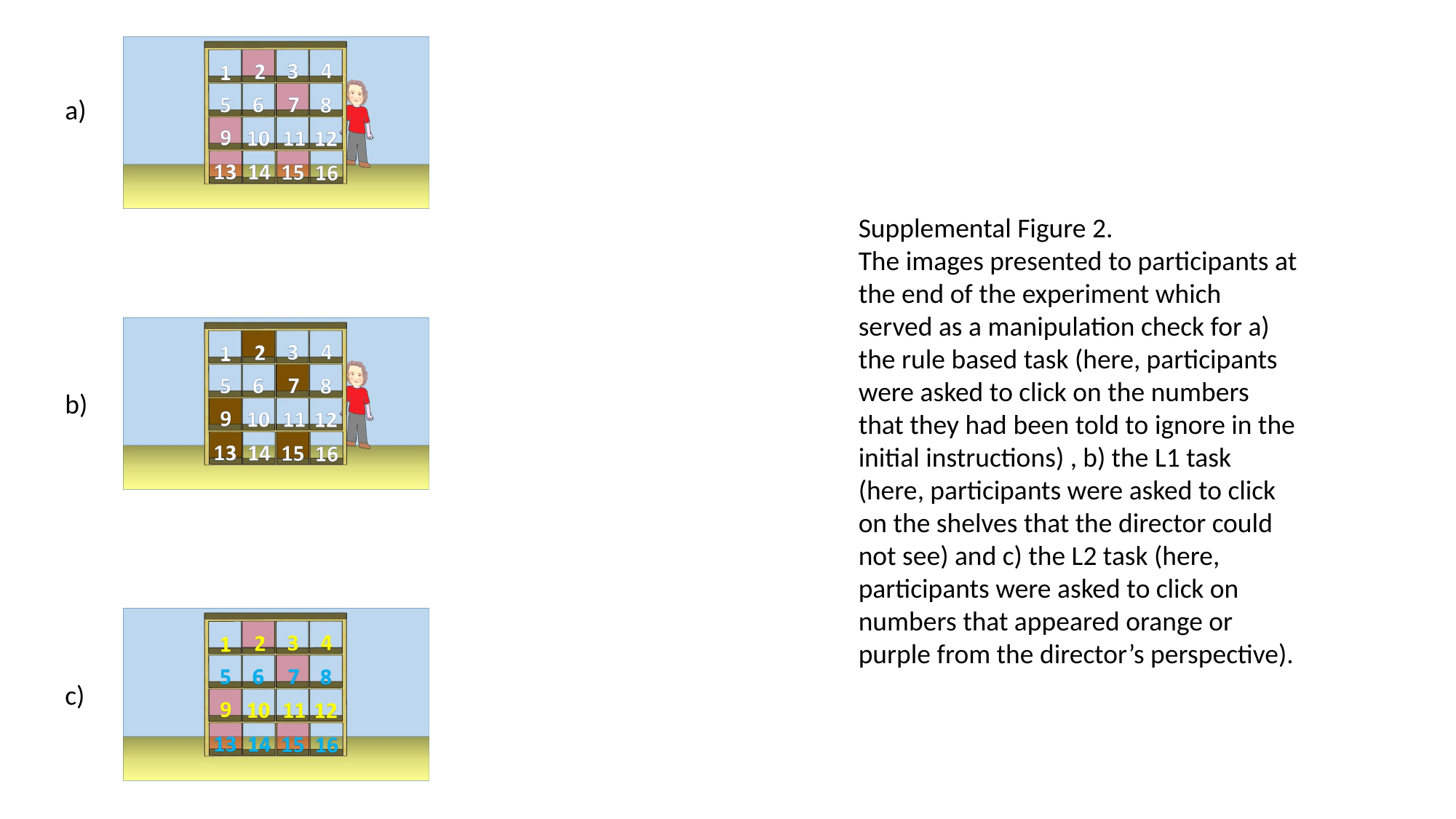

a)
Supplemental Figure 2.
The images presented to participants at the end of the experiment which served as a manipulation check for a) the rule based task (here, participants were asked to click on the numbers that they had been told to ignore in the initial instructions) , b) the L1 task (here, participants were asked to click on the shelves that the director could not see) and c) the L2 task (here, participants were asked to click on numbers that appeared orange or purple from the director’s perspective).
b)
c)
