# Supplementary material for: Error rate on the director's task is influenced by the need to take another's perspective but not the type of perspective
Source: R Soc Open Sci. 2017 Aug 16;4(8):170284. doi: 10.1098/rsos.170284 (PMC5579093; doi:10.1098/rsos.170284)
Supplement: Electronic Supplementary Material [file rsos170284supp2.pdf]

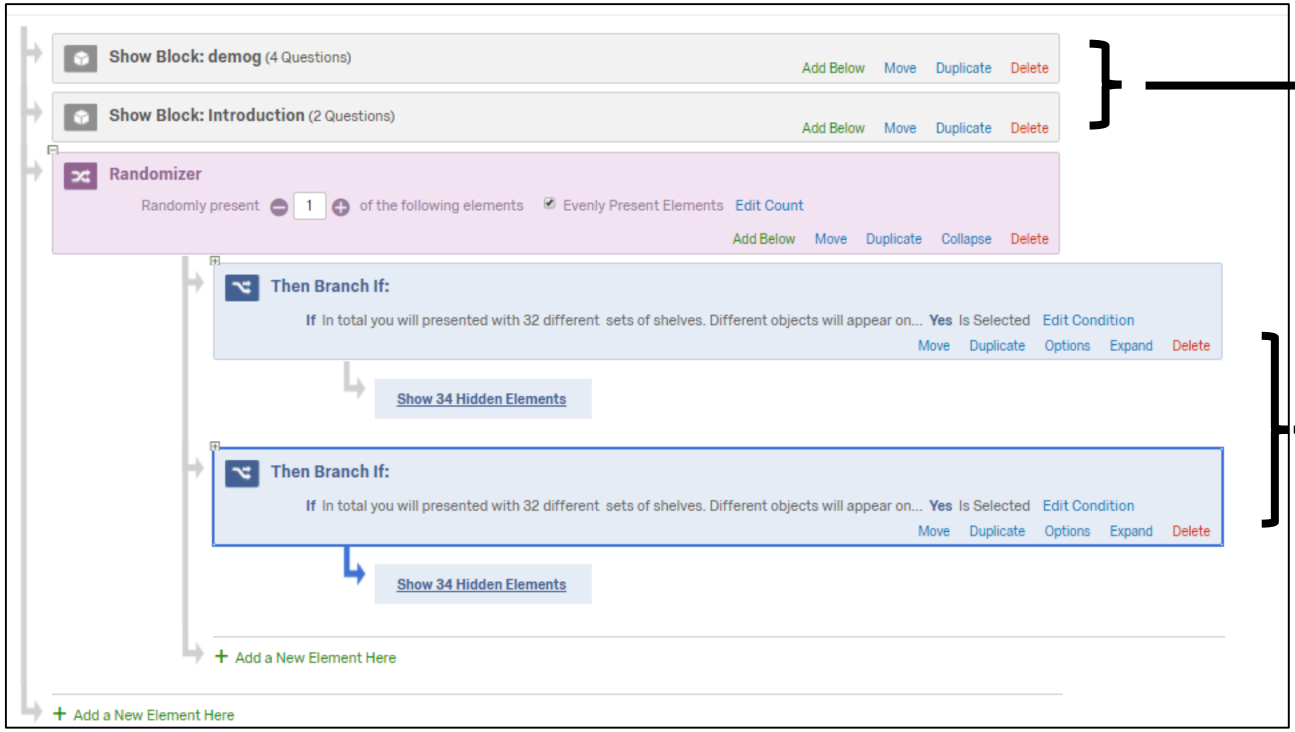

Consent requested and introductory information provided

Participants split into two groups such that half would one trial order and the other the reverse.

The hidden elements are the 32 trials in the experiment. Plus the manipulation check and a debrief form.
